# Contributions of Candida albicans Dimorphism, Adhesive Interactions, and Extracellular Matrix to the Formation of Dual-Species Biofilms with Streptococcus gordonii

**DOI:** 10.1128/mBio.01179-19

**Published:** 2019-06-18

**Authors:** Daniel Montelongo-Jauregui, Stephen P. Saville, Jose L. Lopez-Ribot

**Affiliations:** a Department of Biology, The University of Texas at San Antonio, San Antonio, Texas, USA; b South Texas Center for Emerging Infectious Diseases, The University of Texas at San Antonio, San Antonio, Texas, USA; University of Texas Health Science Center; University of Maryland, Baltimore; University of Connecticut

**Keywords:** *Candida albicans*, *Streptococcus gordonii*, mixed biofilms, synthetic saliva

## Abstract

Fungal and bacterial populations coexist in the oral cavity, frequently forming mixed-species biofilms that complicate treatment against polymicrobial infections. However, despite relevance to oral health, the bidirectional interactions between these microbial populations are poorly understood. In this study, we aimed to elucidate the mechanisms underlying the interactions between the fungal species Candida albicans and the bacterial species Streptococcus gordonii as they coexist in mixed-species biofilms. Specifically, the interactions of different C. albicans mutant strains deficient in filamentation (*efg1*Δ/Δ and *brg1*Δ/Δ), adhesive interactions (*als3*Δ/Δ and *bcr1*Δ/Δ), and production of matrix exopolymeric substances (EPS) (*kre5*Δ/Δ*, mnn9*Δ/Δ, *rlm1*Δ/Δ, and *zap1*Δ/Δ) were evaluated with S. gordonii under different conditions mimicking the environment in the oral cavity. Interestingly, our results revealed that growth of the biofilm-deficient C. albicans *als3Δ/Δ* and *bcr1Δ/Δ* mutant strains in synthetic saliva or with S. gordonii restored their biofilm-forming ability. Moreover, challenging previous observations indicating an important role of morphogenetic conversions in the interactions between these two species, our results indicated a highly synergistic interaction between S. gordonii and the C. albicans filamentation-deficient *efg1Δ/Δ* and *brg1Δ/Δ* deletion mutants, which was particularly noticeable when the mixed biofilms were grown in synthetic saliva. Importantly, dual-species biofilms were found to exhibit increase in antimicrobial resistance, indicating that components of the fungal exopolymeric material confer protection to streptococcal cells against antibacterial treatment. Collectively, these findings unravel a high degree of complexity in the interactions between C. albicans and S. gordonii in mixed-species biofilms, which may impact homeostasis in the oral cavity.

## Abstract (precis)

Microbial communities have a great impact in health and disease. C. albicans interacts with multiple microorganisms in the oral cavity, frequently forming polymicrobial biofilms. We report on the synergistic interactions between C. albicans and the Gram-positive bacterium S. gordonii, for which we have examined the different contributions of adhesive interactions, filamentation, and the extracellular matrix to the formation of dual-species biofilms. Our results demonstrate that growth in the presence of the bacterium can restore the biofilm-forming ability of different C. albicans mutant strains with defects in adhesion and filamentation. The mixed-species biofilms also show high levels of resistance to antibacterial and antifungal antibiotics, and our results indicate that the fungal biofilm matrix protects bacterial cells within these mixed-species biofilms. Our observations add to a growing body of evidence indicating a high level of complexity in the reciprocal interactions and consortial behavior of fungal/bacterial biofilms.

## INTRODUCTION

Candida albicans is commonly found as a normal commensal in the mucosae of humans (1, 2). However, as the main opportunistic fungal pathogen of compromised patients, it has the potential to cause infections, ranging from superficial to invasive disseminated candidiasis (3). The oral cavity is one of the multiple niches where this fungus resides (4), and depending on the host status and environmental conditions, C. albicans can either be a harmless commensal colonizing oral surfaces or lead to active infection, such as oropharyngeal candidiasis, denture stomatitis, or angular cheilitis (5, 6). Interestingly, C. albicans has been reported to interact with other members of the oral microbiota, in particular bacteria, leading to either synergistic or antagonistic interkingdom interactions (7). These interactions often result in the formation of mixed fungal/bacterial biofilms, complex microbial communities displaying consortial behavior, and biofilm formation carries important repercussions to oral health (8–16).

Among the interactions between C. albicans and oral bacteria, those with Streptococcus gordonii are among the best characterized thus far (9, 17–20). This bacterium is considered an early colonizer of the oral cavity, and increasing evidence points to the multiple ways in which these two organisms interact with each other (7, 9, 17, 19, 21). Evidence so far point to the fact that hyphae are the preferred C. albicans morphotype S. gordonii adheres to (17). More specifically, S. gordonii directly binds C. albicans hypha-specific adhesins Als3p, Eap1p, and Hwp1p via the streptococcal SspB, belonging to the antigen I/II family of polypeptides (17, 22). The S. gordonii quorum-sensing factor AI-2 induces C. albicans filamentation, and S. gordonii is able to block the inhibitory effect of exogenous farnesol on C. albicans filamentation and biofilm formation (9). S. gordonii expression of glucosyltransferase G (GtfG) also contributes to coaggregation with C. albicans (23). Also, the S. gordonii *comCDE* (competence) operon modulates the formation of dual-species biofilms with C. albicans (19). In virtually all instances, formation of these mixed fungal/bacterial biofilms led to high levels of antibiotic resistance (20, 24).

In this study, we aimed to elucidate the roles of the key C. albicans biological processes and specific factors during the interactions with S. gordonii in mixed-species biofilms, for which we have used C. albicans mutant deletion strains lacking key specific genes influencing filamentation, adhesive interactions, and/or production of biofilm matrix components. Our combined findings demonstrated a high degree of complexity in the mutualistic interactions between these two microorganisms in dual-species biofilms. Significantly, our findings also indicated a potential therapeutic implication for this interaction, as these diverse species coexist within a biofilm.

## RESULTS AND DISCUSSION

### Growth media impacts the biofilm-forming abilities of various mutant strains of C. albicans.

The use of deletion mutant strains with specific defects in key genes represents a powerful tool for the molecular dissection of biological and developmental processes (25). Therefore, we evaluated the interactions between S. gordonii and various C. albicans mutant strains representing the key fungus-associated factors involved in adherence and biofilm formation, namely, morphogenetic conversions, adhesive interactions, and biofilm polysaccharide matrix (26). Since we have previously demonstrated significant differences in the extent and architectural features between C. albicans biofilms grown in basal medium mucin (BMM) synthetic saliva versus those grown in conventional microbiological media (20), we initially evaluated the biofilm-forming capabilities of the various C. albicans mutant strains in both microbiological media, the BMM synthetic saliva and the 1:1 (vol/vol) mixture of RPMI 1640 and Todd-Hewitt broth (THB) plus 0.02% (wt/vol) yeast extract medium (hereafter referred to as 1:1 media). The mutant strains selected for these experiments (Table 1) are representative of three of the major different categories that play pivotal roles in C. albicans biofilm formation and maintenance, including morphogenetic conversions, adhesive interactions, and the biofilm matrix (26). Since filamentation and biofilm formation are intrinsically linked to each other in this pathogenic fungus (27–30), we chose the *efg1Δ/Δ* and the *brg1Δ/Δ* deletion mutants as archetypical strains with strong filamentation defects, although we note that besides their role in filamentation, *EFG1* and *BRG1* form part of a network of regulators of biofilm formation (29). To understand the contribution of adhesive interactions, the C. albicans *als3Δ/Δ* and *bcr1Δ/Δ* mutants were selected (31). Bcr1p is a master regulator of C. albicans biofilm formation and maintenance due to its control of the expression of key adhesins on the surfaces of fungal cells (32). One of these adhesins and the key target of Bcr1 regulation is Als3, which is expressed specifically on the surfaces of C. albicans hyphae, and Als3-mediated adherence, mostly through complementary interactions with other adhesins, represents a key factor in biofilm formation (18, 33). As a result, biofilm formation is severely impaired in both *bcr1Δ/Δ and als3Δ/Δ* mutant strains, despite the fact that they are fully capable of filamenting. Finally, the *kre5Δ/Δ*, *mnn9Δ/Δ*, *rlm1Δ/Δ*, and *zap1Δ/Δ* mutants were selected on the basis of the important contributions of their corresponding genes/proteins to the C. albicans biofilm matrix (34–36). Fungal cell walls are composed of glucans, mannans, and chitin (37, 38) which also represent major components of the biofilm matrix (35, 39). For these reasons, we tested mutant strains with irregular composition of fungal cell wall (*rlm1Δ/Δ*) (40), reduced amounts of glucans (*kre5Δ/Δ*) (41) or mannans (*mnn9Δ/Δ*) (39, 42, 43) in the cell wall and biofilm exopolymeric material, as well as mutants with alterations in biofilm matrix formation, such as the *zap1Δ/Δ* mutant (44).

**TABLE 1:** C. albicans strains used in this study

C. albicans strain	Reference
SC5314 (parental)	
*als3Δ*/*Δ* mutant	33
*brg1Δ*/*Δ* mutant	55

SN152 (parental)	34
*bcr1Δ*/*Δ* mutant	34
*efg1Δ*/*Δ* mutant	34
*kre5Δ*/*Δ* mutant	35
*mnn9Δ*/*Δ* mutant	35
*rlm1Δ*/*Δ* mutant	34
*zap1Δ*/*Δ* mutant	34

**FIG 1:**
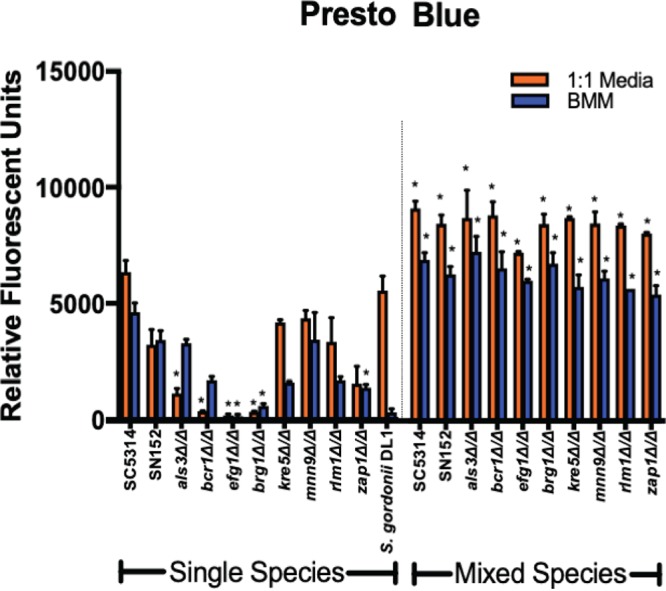
Extent of biofilm formation of single-species biofilms formed by the different C. albicans parental and deletion mutant strains and mixed-species biofilms formed with the same strains together with S. gordonii. Biofilms were grown in 1:1 media (1:1 [vol/vol] RPMI 1640 and THB plus 0.02% YE) or BMM synthetic saliva (BMM) in 96-well microtiter plates. Viability of cells within the biofilms was measured by Presto Blue fluorescence. Data represent at least two independent experiments with three replicates per sample. Error bars represent standard errors of the means. Values that are statistically significantly different from the values for the corresponding parental strains (*P < *0.05) are indicated by an asterisk.

**FIG 2:**
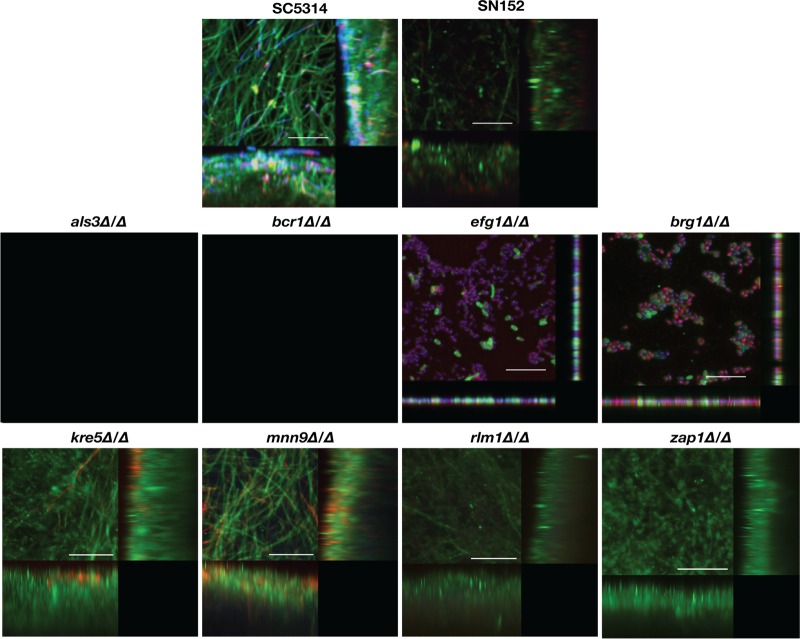
CSLM of C. albicans single-species biofilms formed by the different parental and deletion mutant strains grown in 1:1 media. Biofilms were stained using concanavalin A–Alexa Fluor 488 fungal cell wall stain (green), DAPI nucleic acid stain (blue), and FilmTracer Sypro Ruby biofilm matrix stain (red) and observed using a ×63 oil objective. Bars are 50 μm for all panels. Included in the figure are the *xy*, *xz*, and *yz* views of the corresponding biofilms.

Similar to what has been previously described in the literature of C. albicans mutant strains (18, 31, 32, 45–47), the data obtained from Presto Blue metabolic readings (Fig. 1) pointed to defects in the biofilm-forming ability associated with some of the tested mutant strains in comparison to their respective parental strains (SC5314 for the *als3Δ/Δ* and *brg1Δ/Δ* mutant strains, SN152 for all other strains tested) when using traditional microbiological media to form monospecies biofilms (Fig. 1). This was particularly noticeable in the case of those mutant strains with defects in adhesive interactions (*als3Δ/Δ* and *bcr1Δ/Δ*), and filamentation-defective mutant strains (*efg1Δ/Δ* and *brg1Δ/Δ*), whereas no major differences were observed in the case of mutant strains carrying deletions affecting key components of the biofilm exopolymeric matrix (i.e., *kre5Δ/Δ* and *mnn9Δ/Δ*). These observations were further corroborated by visualization of the resulting biofilms using confocal scanning laser microscopy (CSLM) as shown in Fig. 2 and by scanning electron microscopy (SEM) as shown in Fig. 6 and Fig. S1 in the supplemental material. Similar to what has previously been described using different microbiological media (29, 46), the filamentation-deficient C. albicans *efg1Δ/Δ* mutant strain was severely deficient in biofilm formation when 1:1 media were used, forming a thin layer of mostly elongated yeast cells, and very similar observations were made in the case of the *brg1Δ/Δ* mutant (Fig. 2 and Fig. S1). In 1:1 media, the biofilms formed by C. albicans *als3Δ/Δ* and *bcr1Δ/Δ* mutants with defects in adhesive interactions were fragile and readily detached from the plate surface when we attempted to stain for confocal microscopy or scanning electron microscopy (we note that taking extreme caution during the washing/fixing/dehydration processing of samples for SEM, we were able to retain a small portion of the *bcr1Δ/Δ* biofilm, which is included in Fig. 6 and Fig. S1), also similar to previous observations for *in vitro*-formed biofilms by these strains (29).

10.1128/mBio.01179-19.1 FIG S1 SEM observations of C. albicans single-species and C. albicans/S. gordonii mixed-species biofilms formed by the different parental and deletion mutant strains. These images are lower-magnification images (compared to those shown in Fig. 6) to display a wider field of observation. Magnification is ×500, and the scale bars are 50 μm for all panels, except for the *bcr1Δ/Δ* monospecies biofilm in 1:1 media, which was observed at a magnification of ×50, and the scale bar represents 500 μm. Download FIG S1, PDF file, 0.2 MB. Copyright © 2019 Montelongo-Jauregui et al. 2019 Montelongo-Jauregui et al. This content is distributed under the terms of the Creative Commons Attribution 4.0 International license.

**FIG 3:**
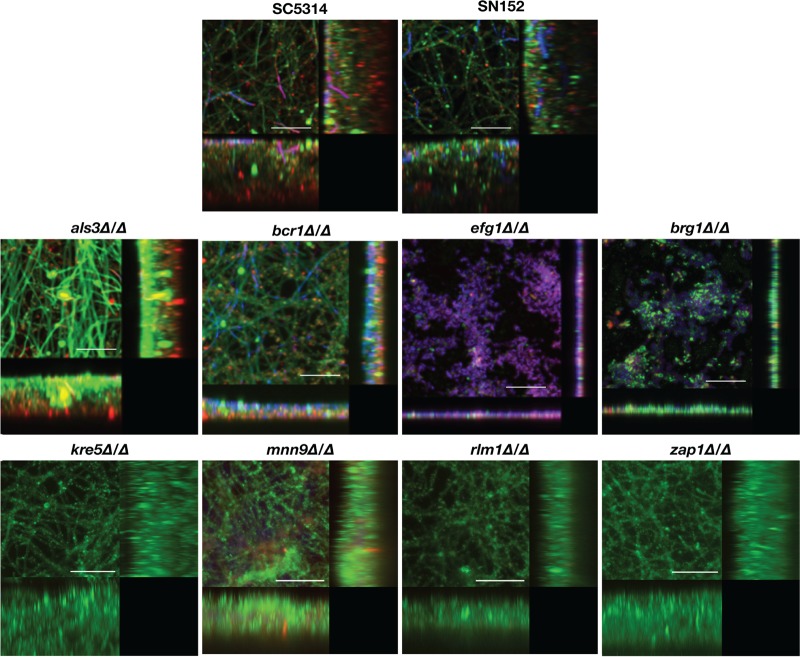
CSLM of C. albicans single-species biofilms formed by the different parental and deletion mutant strains grown in BMM synthetic saliva. Biofilms were stained using concanavalin A–Alexa Fluor 488 fungal cell wall stain (green), DAPI nucleic acid stain (blue), and FilmTracer Sypro Ruby biofilm matrix stain (red) and observed using a ×63 oil objective. Bars are 50 μm for all panels. Included in the figure are the *xy*, *xz*, and *yz* views of the corresponding biofilms.

In contrast to the significant deficiencies in biofilm formation in the 1:1 media, interestingly, in BMM synthetic saliva, both the C. albicans *als3Δ/Δ* and *bcr1Δ/Δ* deletion mutants were capable of forming biofilms in BMM synthetic saliva to an extent similar to those formed by their corresponding parental strains, as manifested by increased Presto Blue metabolic readings (Fig. 1), as well as CSLM (Fig. 3) and SEM visualizations (see Fig. 6 and Fig. S1). As mentioned before, this synthetic saliva medium mimics *in vivo* conditions in the mouth, which may explain the differences observed between BMM and 1:1 media. This is also in agreement with previous reports indicating that the *als3Δ/Δ* mutant strain is capable of forming biofilms *in vivo* in a catheter model (31, 32). However, we note that growth in BMM synthetic saliva did not restore the biofilm-forming ability of the filamentation-deficient *efg1Δ/Δ* and *brg1Δ/Δ* deletion mutant strains (see also Fig. 3 and Fig. S1).

### Examination of different C. albicans mutant strains indicates that morphology, adherence, and extracellular matrix make distinct contributions to the formation of mixed-species biofilms with S. gordonii.

As mentioned before, C. albicans and S. gordonii are often found in association with each other as members of the human oral microbiota (48, 49). Together, they have the ability to form dual-species biofilms that may impact their roles as commensals and pathogenic microorganisms of the oral cavity of humans (48). Results thus far point to a key role of filamentation and adherence as the two major factors mediating this mutualistic beneficial relationship (50). Although these interactions are clearly bidirectional, a majority of studies have come from the bacterial aspect (9, 19, 23).

**FIG 4:**
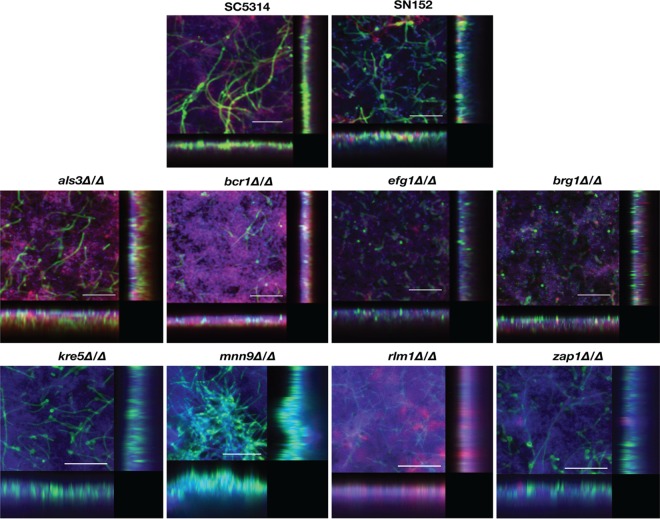
CSLM of C. albicans/S. gordonii dual-species biofilms formed by the different parental and deletion mutant strains grown in 1:1 media. Biofilms were stained using concanavalin A–Alexa Fluor 488 fungal cell wall stain (green), DAPI nucleic acid stain (blue), and FilmTracer Sypro Ruby biofilm matrix stain (red) and observed using a ×63 oil objective. Bars are 50 μm for all panels. Included in the figure are the *xy*, *xz*, and *yz* views of the corresponding biofilms.

**FIG 5:**
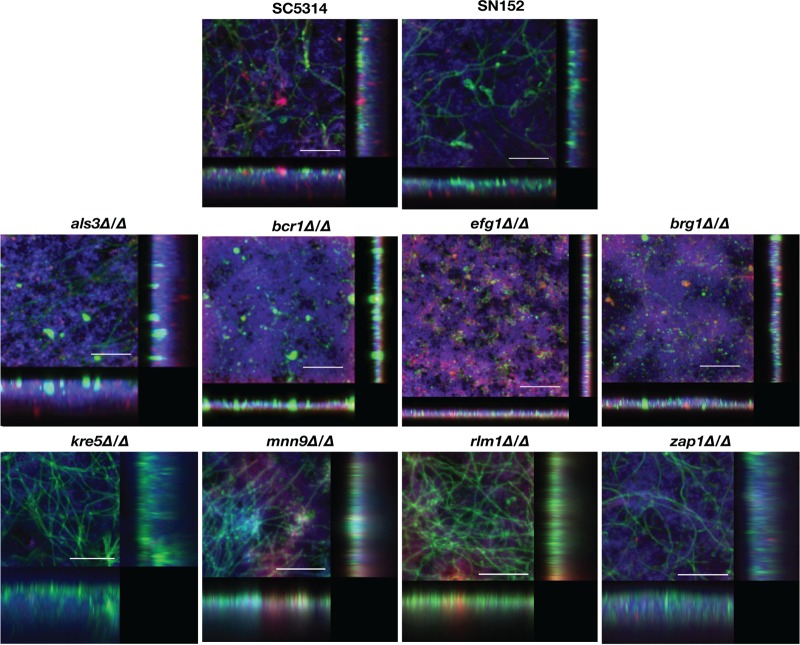
CSLM of C. albicans/S. gordonii dual-species biofilms formed by the different parental and deletion mutant strains grown in BMM synthetic saliva. Biofilms were stained using concanavalin A–Alexa Fluor 488 fungal cell wall stain (green), DAPI nucleic acid stain (blue) and FilmTracer Sypro Ruby biofilm matrix stain (red) and observed using a ×63 oil objective. Bars are 50 μm for all panels. Included in the figure are the *xy*, *xz*, and *yz* views of the corresponding biofilms.

**FIG 6:**
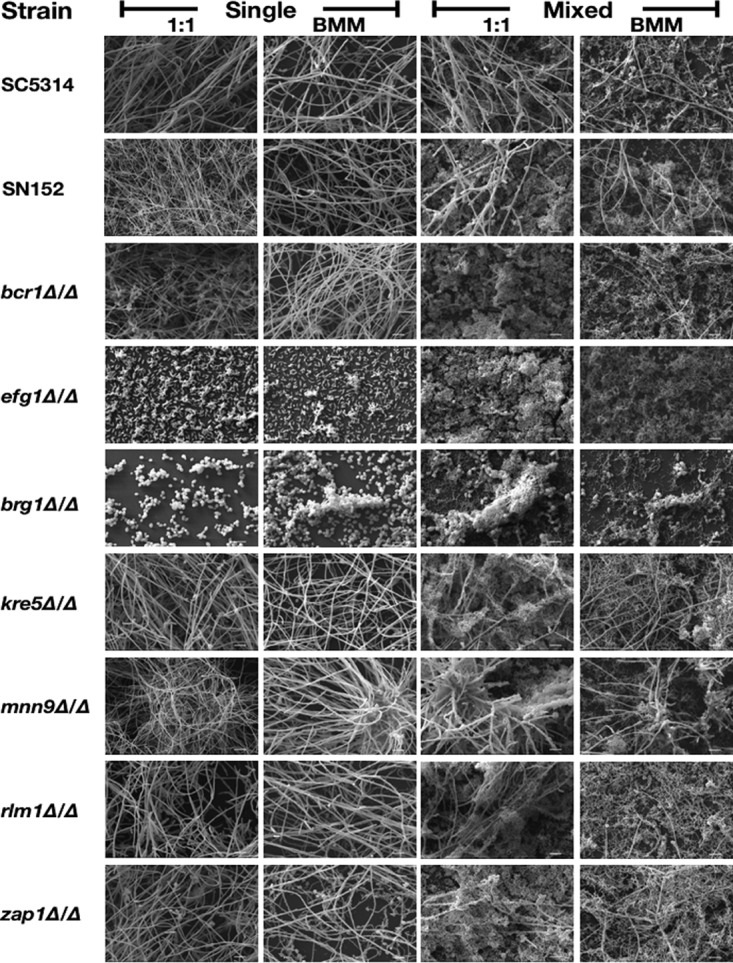
SEM observations of C. albicans single-species and C. albicans/S. gordonii mixed-species biofilms formed by the different parental and deletion mutant strains. Magnification is ×1,000. Bars, 10 μm.

Previous studies have clearly indicated that S. gordonii and C. albicans interactions occur preferentially with hyphal elements compared to yeast cells (9, 17, 20). Thus, we were interested in examining the interactions between S. gordonii and C. albicans *efg1Δ/Δ* and *brg1Δ/Δ* mutants, which are unable to filament under most conditions tested, in turn exhibiting severe defects in the formation of single-species biofilms as previously reported (46) and shown above (Fig. 1 and 3). However, in our experiments and somewhat surprisingly, we observed that these C. albicans mutant strains formed dual-species biofilms to an extent similar to that of their parental strain. This was manifested by results of Presto Blue viability readings to assess the extent of dual-species biofilm formation as seen in Fig. 1 (also confirmed by CFU counts of bacterial and fungal cells in mixed-species biofilms [Fig. S2]), both in 1:1 media and BMM synthetic saliva. Results clearly indicated a high level of synergism in the interactions between both filamentation-deficient mutants and S. gordonii, with viability readings of dual-species biofilms being much higher than those observed for monospecies biofilms, which was particularly noticeable in the case of BMM synthetic saliva (compare the values for dual-species biofilms to the values for their corresponding monospecies biofilms formed by each fungal or bacterial strain alone). Moreover, the extent of biofilm formation in dual-species biofilms formed by *efg1Δ/Δ* and *brg1Δ/Δ* strains together with S. gordonii were similar to those formed by their parental strains (Fig. 1). Using CSLM, dual-species biofilms formed by either the *efg1Δ/Δ* or the *brg1Δ/Δ* mutant strains together with S. gordonii demonstrated increased density and thickness compared to their corresponding monospecies biofilms (Fig. 4 and 5). SEM observations confirmed these results and also demonstrated the tight and direct binding of bacterial cells to yeast cells of both the C. albicans *efg1Δ/Δ* and *brg1Δ/Δ* strains (Fig. 6). Our results demonstrate that the presence of S. gordonii can restore biofilm formation of filamentation-defective C. albicans mutant strains, and altogether indicate that, contrary to what was previously thought, filamentation is not an absolute requirement for the formation of dual-species biofilms of C. albicans with S. gordonii.

10.1128/mBio.01179-19.2 FIG S2 Determination of fungal and bacterial CFUs from single- and mixed-species biofilms formed by the different parental and deletion mutant strains. C. albicans CFUs were recovered by plating cells recovered from biofilms on YPD agar with antibiotics (5 mg/ml penicillin, 5 mg/ml streptomycin, 10 mg/ml neomycin), whereas S. gordonii CFUs were recovered by plating contents on tryptone soy agar with 5% sheep blood plates with 4 μg/ml amphotericin B. Download FIG S2, PDF file, 0.2 MB. Copyright © 2019 Montelongo-Jauregui et al. 2019 Montelongo-Jauregui et al. This content is distributed under the terms of the Creative Commons Attribution 4.0 International license.

The direct binding between C. albicans Als3 and S. gordonii SspB belonging to the antigen I/II family of polypeptides plays a key role in the coaggregation between these two microorganisms and as such has been characterized in depth at the molecular level (22). Streptococcal cells failed to attach to filaments of the C. albicans *als3Δ/Δ* mutant strain, and the same group also reported that Als3 is necessary for the formation of dual-species biofilms with S. gordonii (17, 22). However, in our experiments, we were able to demonstrate that cells of the C. albicans *als3Δ/Δ* mutant strain could incorporate into biofilms together with S. gordonii, resulting in the formation of mixed fungal/bacterial biofilms that were comparable to those formed by its corresponding wild-type parental strain SC5314, as estimated by Presto Blue metabolic readings (Fig. 1), and corroborated by CFU analyses of cells recovered from the biofilms (Fig. S2), as well as CSLM visualization of the resulting dual-species biofilms (Fig. 4 and 5), both using 1:1 media (in which monospecies biofilms formed by this mutant strain readily detach from these surfaces [see above]) and BMM synthetic saliva. As mentioned above, this is in stark contrast to previous reports (17). We posit that differences in the experimental design may account for the disparate results between our current study and those previous reports. For example, Silverman et al. used a model in which cells of the *als3Δ/Δ* mutant were added to a preformed monolayer of streptococci and reported that they were incapable of first binding and subsequently forming a biofilm (17). Our experimental design was different; wells of the microtiter plates were seeded at the same time with both fungal and bacterial cells, and dual-species biofilms were then able to form upon subsequent incubation over 24 h. It is also possible that the media used in our studies also favor the formation of these mixed biofilms: use of nutritionally rich 1:1 media supports equal growth of both fungal and bacterial cells (20), whereas use of BMM synthetic saliva more closely resembles physiological conditions within the oral cavity (20, 24, 51) and, as demonstrated above, can restore the biofilm-forming ability of this C. albicans mutant in monospecies biofilms. We extended our observations to the C. albicans *bcr1Δ/Δ* mutant strain. Bcr1p is a major regulator of C. albicans biofilm formation since it controls the expression of key adhesins on the surfaces of fungal cells (32). As such, cells of the *bcr1Δ/Δ* mutant, despite being able to filament, not only lack expression of Als3 but also expression of other adhesins such as Als1p, as well as Hwp1p (32). Although capable of filamenting, the C. albicans *Δbcr1* deletion mutant strain shows a dramatic biofilm defect (52). Despite this, and similar to the results using the *als3Δ/Δ* mutant, cells of the C. albicans *bcr1Δ/Δ* mutant were able to incorporate into dual-species biofilms with S. gordonii. The extent of biofilm formation in dual-species biofilms formed by the *bcr1Δ/Δ* mutant with S. gordonii was similar to that observed for the parental strain (Fig. 1), with detection of both fungal and bacterial cells in the biofilms as demonstrated by CFU analyses (Fig. S2). As shown in Fig. 2, 4, and 6, CSLM and SEM demonstrated that in contrast to monospecies biofilms formed by the same mutant in 1:1 media that readily detach from the surface and wash away, the dual-species biofilms formed by the C. albicans *bcr1Δ/Δ* deletion strain were robust and display features similar to biofilms formed by the parental strain, including filamentation and extensive interactions between fungal and bacterial cells, and a similar overall architecture. Thus, it would seem that although Als3 on the surfaces of C. albicans filaments, and possibly other Bcr1p-regulated adhesins, may represent major receptors for the attachment of bacterial cells to C. albicans hyphae mostly through their direct interactions with S. gordonii SspB (17), other adhesive interactions can occur between fungal and bacterial cells that still allow for the formation of dual-species biofilms even in the absence of these major adhesins. Interestingly, these results are similar to those reported recently showing that a C. albicans *bcr1Δ/Δ* mutant strain was able to form dual-species biofilms with Streptococcus mutans (53).

As expected by their ability to form monospecies biofilms, C. albicans mutant strains with deletions of cell wall/biofilm matrix component genes *kre5Δ/Δ*, *mnn9Δ/Δ, rlm1Δ/Δ*, and *zap1Δ/Δ* did not display major defects in the formation of dual-species biofilms with S. gordonii. As shown in Fig. 1, mixed-species biofilms formed by these mutants had similar Presto Blue metabolic readings compared to their parental strain. CSLM and SEM observations showed that these biofilms displayed morphological (i.e., presence of hyphae) and architectural features (density and thickness) similar to those formed by the C. albicans SN152 strain (Fig. 4 and 6).

A summary of observations on the biofilm-forming ability and structural characteristics of biofilms formed by the different C. albicans mutant strains under the different growing conditions employed in these experiments is shown in Table 2.

**TABLE 2:** Summary of observations of biofilms formed by the different C. albicans mutant strains under different growth conditions compared to their corresponding parental strains^*a*^

C. albicans mutant strain	Single-species biofilms		Dual-species biofilms with S. gordonii^*b*^
	1:1 media	BMM	
*als3****Δ*/*Δ***	Detached upon washing	Normal	Normal
*bcr1****Δ*/*Δ***	Detached upon washing	Normal	Normal
*efg1****Δ*/*Δ***	Yeast cell monolayer	Yeast cell monolayer	Yeast cells incorporate
*brg1****Δ*/*Δ***	Yeast cell monolayer	Yeast cell monolayer	Yeast cells incorporate
*kre5****Δ*/*Δ***	Normal	Normal	Normal
*mnn9****Δ*/*Δ***	Normal	Normal	Normal
*rlm1****Δ*/*Δ***	Normal	Normal	Normal
*zap1****Δ*/*Δ***	Normal	Normal	Normal

a Summary of observations on the biofilm-forming ability and structural characteristics of biofilms formed by the different C. albicans mutant strains under different growth conditions compared to their corresponding parental strains.

b Observations for dual-species biofilms in the presence of S. gordonii were generally similar for biofilms grown on 1:1 media and BMM synthetic saliva.

### Antimicrobial susceptibility patterns of dual-species biofilms formed by the different C. albicans mutant strains indicate a protective role of components of the fungal matrix.

**FIG 7:**
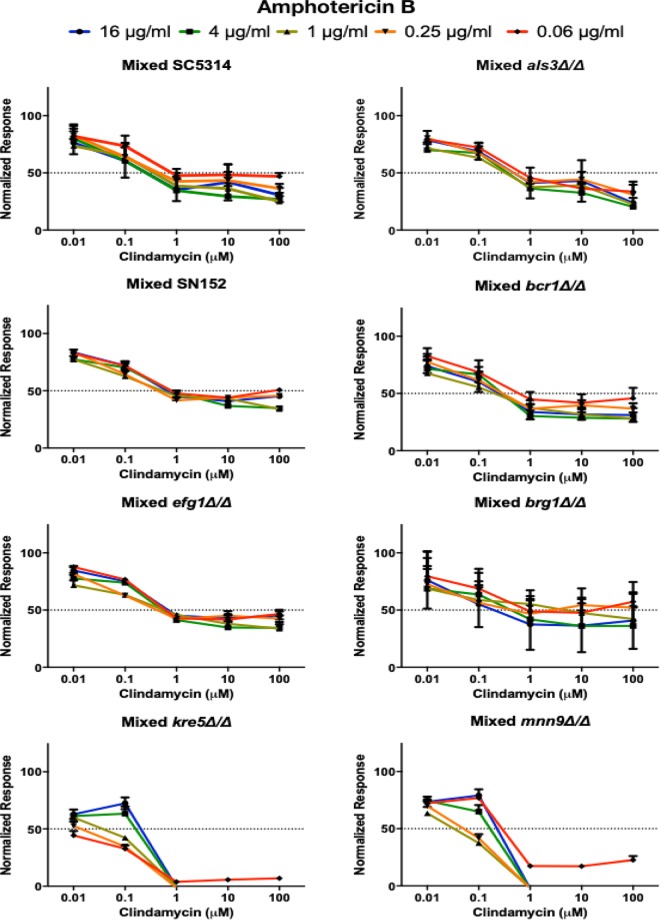
Effect of combination therapy against mixed-species biofilms. Antimicrobial susceptibility testing was performed by combining drugs (clindamycin plus amphotericin B) and adding to 24-h preformed C. albicans/S. gordonii mixed-species biofilms formed by the different parental and deletion mutant strains in 1:1 media. Drugs were added at the following concentrations: clindamycin at 100, 10, 1, 0.1, and 0.01 μM and amphotericin B at 16, 4, 1, 0.25, and 0.06 μg/ml. Error bars represent standard errors of the means.

**FIG 8:**
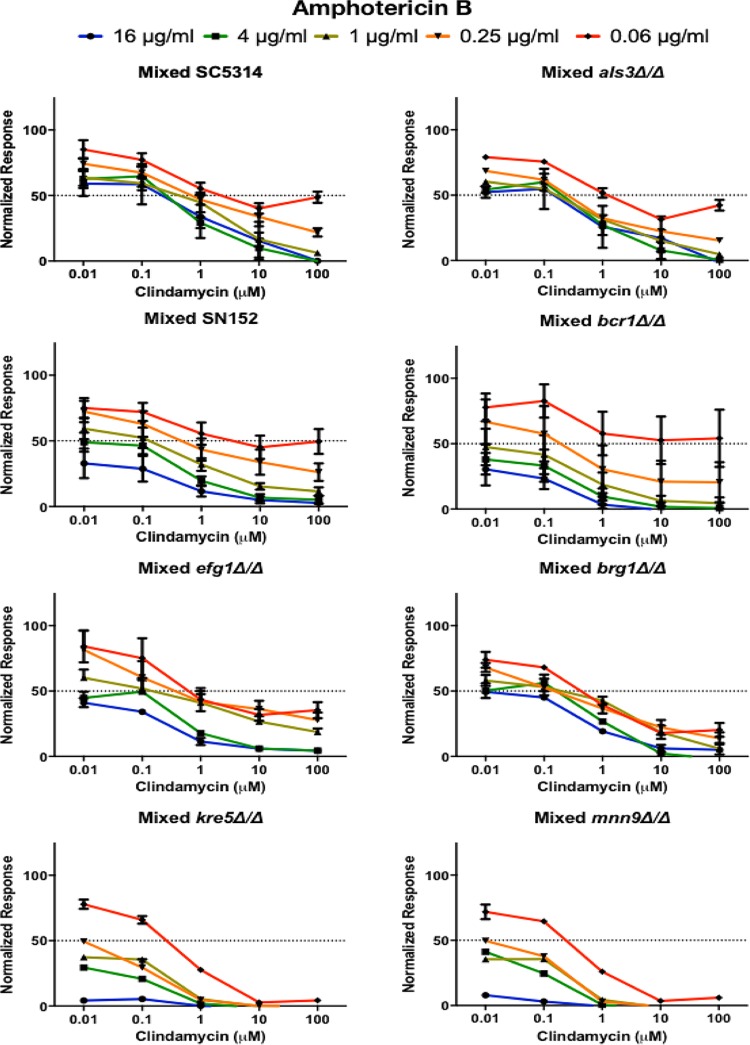
Effect of combination therapy against mixed-species biofilms. Antimicrobial susceptibility testing was performed by combining drugs (clindamycin plus amphotericin B) and adding to 24-h preformed C. albicans/S. gordonii mixed-species biofilms formed by the different parental and deletion mutant strains in BMM synthetic saliva. Drugs were added at the following concentrations: clindamycin at 100, 10, 1, 0.1, and 0.01 μM and amphotericin B at 16, 4, 1, 0.25, and 0.06 μg/ml. Error bars represent standard errors of the means.

Our previous studies demonstrated high level of resistance for preformed mixed biofilms of C. albicans and S. gordonii (20, 24), against antifungals and the antibacterial clindamycin, both in monotherapy and in combinatorial treatment (20, 24). In order to provide some mechanistic insights into these phenomena, we assessed the susceptibility patterns of dual-species biofilms formed by S. gordonii and the various C. albicans deletion mutant strains. Results from these assays demonstrated that monotherapy with either clindamycin or amphotericin B was highly ineffective against mixed biofilms formed by the C. albicans *als3Δ/*Δ, *bcr1Δ/*Δ, *efg1Δ/*Δ, and *brg1*Δ*/*Δ deletion mutant strains both in 1:1 media and in BMM synthetic saliva (Fig. S3 and S4). Interestingly, although S. gordonii biofilms with the *kre5Δ/Δ* and *mnn9Δ/Δ* mutant strains were similarly resistant to amphotericin B, they exhibited enhanced susceptibility to clindamycin, indicating that C. albicans matrix components protect S. gordonii cells from antimicrobial treatment, similar to previous observations in mixed biofilms with other bacterial species (36, 54). Subsequently, we performed combination treatment with both amphotericin B and clindamycin against preformed dual-species biofilms. Results from these experiments indicated that mixed biofilms with the C. albicans adhesin and filamentation deletion mutant strains (*als3*Δ/Δ, *bcr1*Δ/Δ, *efg1*Δ/Δ, and *brg1Δ/Δ*) displayed high levels of resistance to antimicrobial treatment comparable to those formed by their respective parental strains (Fig. 7 and 8). Interestingly, mixed biofilms formed in BMM were generally more susceptible against combination treatment than biofilms formed by the same strains in 1:1 media. Consistent with findings from monotherapy experiments, preformed mixed-species biofilms formed by S. gordonii together with the C. albicans *kre5Δ/Δ* and *mnn9Δ/Δ* deletion mutant strains displayed higher susceptibility to combinatorial treatment, again pointing to a crucial role for the C. albicans cell wall-secreted carbohydrates (glucans and mannans) that form the biofilm matrix in conferring coexisting S. gordonii with protection against antimicrobials (Fig. 7 and 8). Although the effect of fungal exopolymeric matrix has not been previously reported to protect S. gordonii, these results are similar to those observed in dual-species biofilms formed by C. albicans together with Staphylococcus aureus, as elegantly demonstrated by the Jabra-Rizk group (54). Of note, the reciprocal observation has also been reported in the case of mixed biofilms with S. mutans, where the bacterial exopolymeric substance was shown to protect the C. albicans cells from treatment with antifungals (36).

10.1128/mBio.01179-19.3 FIG S3 Antimicrobial susceptibility patterns of preformed C. albicans/S. gordonii mixed-species biofilms formed by the different parental and deletion mutant strains in 1:1 media using monotherapy with either amphotericin B or clindamycin. Drugs were added at the following concentrations: clindamycin at 100, 10, 1, 0.1, and 0.01 μM and amphotericin B at 16, 4, 1, 0.25, and 0.0625 μg/ml. Error bars represent standard errors of the means. Download FIG S3, PDF file, 0.2 MB. Copyright © 2019 Montelongo-Jauregui et al. 2019 Montelongo-Jauregui et al. This content is distributed under the terms of the Creative Commons Attribution 4.0 International license.

Combined, our results demonstrate the high degree of mutualistic cooperation and a high level of complexity in the formation of dual-species biofilms by C. albicans and S. gordonii. For example, growth in BMM synthetic saliva, as well as coculture with S. gordonii, rescues the biofilm-forming defects of the C. albicans *als3Δ/Δ* and *bcr1Δ/Δ* deletion mutant strains normally associated with the absence of these critical adhesins. Moreover, our results using the C. albicans *efg1Δ/Δ* and *brg1Δ/Δ* mutant strains indicate that filamentation is not an absolute requirement for the formation of dual-species biofilms between these two microorganisms. We also note that our results do not negate a role for each of these important biological processes and individual factors in the fungal/bacterial interaction as previously reported by other groups; rather, they point to the complexity of this relationship and seem to indicate the existence of redundant/compensatory mechanisms for each of these interactions between C. albicans and S. gordonii during the formation of dual-species biofilms. Furthermore, although these interactions still occur even with reduced presence of glucans and mannans in the fungal cell walls and extracellular matrices, results from susceptibility testing also point to the fact that that these fungal matrix components can protect bacterial cells in mixed biofilms.

## MATERIALS AND METHODS

### Strains and growth conditions.

S. gordonii wild-type strain Challis DL1.1 was used in all experiments. The C. albicans strains used in this study are provided in Table 1. These strains included C. albicans wild-type strain SC5314 and strain SN152 (34), which is the parental strain for the all homozygous mutant deletion strains used in this study with the exception of the *als3Δ/Δ* and the *brg1Δ/Δ* deletion mutant strains, which are in the SC5314 background (33).

C. albicans strains were cultured on yeast peptone dextrose (YPD) agar plates at 30°C. C. albicans suspension cultures were grown in 20 μl of YPD medium in an orbital shaker at 28°C overnight. Cells were harvested by centrifugation, washed in PBS, and resuspended in the desired media for biofilm growth. A final suspension of 1 × 10^6^ cells/ml in the corresponding medium was seeded for biofilm formation in the wells of microtiter plates (see below). S. gordonii was regularly cultured on tryptone soy agar with 5% sheep blood plates, inside a CO_2_ incubator. Suspension cultures of S. gordonii were grown in 20 ml of Todd-Hewitt broth plus 0.02% (wt/vol) yeast extract (THB plus 0.02% YE) media, without shaking inside a 5% CO_2_ incubator for 16 h at 37°C. After incubation, 100 μl from the suspension culture was aspirated and inserted into 10 ml of fresh THB plus 0.02% YE media and shaken in an orbital shaker (150 to 180 rpm) for 3 h at 37°C. Bacterial cells were then harvested by centrifugation and washed with PBS. Bacterial cell concentrations were calculated measuring OD_600_ with a spectrophotometer. Dilutions were performed in order to obtain a final concentration of 1.0 × 10^7^ cells/ml in the desired media for growth of biofilms (see below).

### Biofilm formation.

Formation of monospecies and dual-species biofilms was done mostly as previously described by our group using 96-well microtiter plate models with slight modifications (20). Briefly, bacterial and fungal cell seeding was done by adding 100 μl per well of the prepared single or mixed cell suspensions in the appropriate medium. The final cell concentrations were C. albicans at 1 × 10^6^ cell/ml and S. gordonii at 1 × 10^7^ cell/ml. After seeding, the 96-well plates (Corning Incorporated, Corning, NY, USA) were incubated in a 5% CO_2_ incubator for 24 h at 37°C. Presto Blue Cell Viability Reagent (Invitrogen, Carlsbad, CA) was used for estimation of biofilm formation using a microtiter plate reader (BioTek Synergy HT, Winooski, VT) to measure fluorescence at 530/25 nm excitation and 590/35 emission.

Biofilms were formed using two different types of microbiological media, as previously described by our group (20). The first one was an equal part mixture of RPMI 1640 and THB plus 0.02% YE media, as we have previously demonstrated that this combination of media supports the growth of both microorganisms (51); while the second one was a BMM synthetic saliva medium that more closely resembles physiological conditions within the oral cavity (20). Preparation of BMM synthetic saliva followed the method described by Wong and Sissons (51), and consists of the following: 2.5 g partially purified pig gastric mucin, 5 g protease peptone (PP), 5 g yeast extract (YE), 33.5 mmol KCl, 2.5 mg hemin, 1 mg menadione, 1 mmol urea, and 1 mmol arginine, diluted in a liter of Millipore water and sterilized in an autoclave.

### Visualization of biofilms by scanning electron microscopy and confocal scanning laser microscopy.

Scanning electron microscopy (SEM) and confocal scanning laser microscopy (CSLM) to visualize monospecies and dual-species biofilms were performed as previously described by our group (20). Briefly, for SEM, biofilms were grown on 6-well plates and fixed with a solution of 2.5% (wt/vol) glutaraldehyde−0.1 M sodium cacodylate buffer at pH 7.4 for 2 h at 37°C. Following fixation, the samples were treated with 1% (wt/vol) osmium tetroxide solution−0.1 M sodium cacodylate buffer at pH 7.4 for 2 h at room temperature. Samples were then rinsed with water and washed in a graded series of ethanol solutions (a step gradient of 30%, 50%, 70%, and 90% ethanol in water for 10 min per step) ending with 100% ethanol. Samples were then dried overnight in a vacuum dryer and subsequently coated with a 60:40 gold-palladium alloy, with an approximate thickness of 883 Å using a sputter coater. Samples were visualized using a JEOL JSM-6610 scanning electron microscope (JEOL USA, Inc., Peabody, MA).

For CSLM, biofilms were stained in the dark in the following order: at 37°C for 30 min with 25 μg/ml concanavalin A–Alexa Fluor 488 conjugate (Molecular Probes, Eugene, OR), at room temperature for 30 min with 1× FilmTracer Sypro Ruby biofilm matrix stain (Molecular Probes), and for 10 min at 37°C with 300 nM 4’,6-diamidino-2-phenylindole, dihydrochloride (DAPI) (Molecular Probes). After incubation, the supernatant was removed, and the biofilms were rinsed two times with sterile PBS to remove nonadhered cells. Samples were viewed using a LSM 510 upright confocal microscope (Carl Zeiss, Thornwood, NY) with an Achroplan 63× oil objective, using excitation/emission wavelengths of 358/461 nm for blue fluorescence, 495/519 nm for green fluorescence, and 450/610 nm for red fluorescence. Pictures were analyzed using AutoQuant X2 (Media Cybernetics, Rockville, MD). Microscopy images were processed for display using Photoshop software (Adobe, Mountain View, CA).

### Comparative microbial recovery from biofilms.

Biofilms grown in 96-well plates were scraped off the bottom of wells, and contents were diluted in sterile PBS, sonicated, and vortexed vigorously. C. albicans CFU were recovered by plating biofilm contents on YPD agar with antibiotics 1× PSN by Gibco (Life Technologies, Carlsbad, CA). S. gordonii CFU were recovered by plating contents on tryptone soy agar with 5% sheep blood plates with 4 μg/ml amphotericin B.

### Susceptibility testing of cells within biofilms.

Drugs were added at desired concentrations to preformed mono- and dual-species biofilms grown in different media (1:1 media or BMM synthetic saliva). Amphotericin B was obtained in solution at 250 μg/ml (Gibco Life Technologies, Grand Island, NY) and stored at −20°C until used. Clindamycin (RPI Corp., Prospect, IL) was obtained as a powder, and stock solutions were stored at 4°C until used. The drugs and final concentrations tested were as follows: amphotericin B at 16, 4, 1, 0.25, 0.06 μg/ml and clindamycin at 100, 10, 1, 0.1, 0.01 μM. After incubation for an additional 24 h, microtiter plates were washed and processed using the Presto Blue assay as described above.

### Statistics.

Viability assays of single- and dual-species biofilms were performed two times with at least three replicates for each growth condition assessed. The data were analyzed using Prism (GraphPad, La Jolla, CA), and the differences were considered statistically significant if *P* < 0.05 by two-way ANOVA test. Dunnett’s multiple comparison was performed using single-species biofilms of strain SC5314 (for the *als3Δ/Δ* and *brg1Δ/Δ* mutants) and SN512 (for all other C. albicans mutant strains), and differences were considered statistically significant if *P* < 0.05. Drug susceptibility assays were performed in triplicate, data were normalized with respect to the average for three positive-control samples (no-drug sample) set at 100%, and the average for three negative-control samples (treated with 10% Triton X-100), set at 0%. For each sample, the average for three replicates was subtracted by the average for the positive controls and divided by the difference of positive and negative controls, and finally multiplied by 100, or [(sample average − POS average)/(POS average − NEG average)] × 100 where POS average is the average for positive controls and NEG average is the average for negative controls.

10.1128/mBio.01179-19.4 FIG S4 Antimicrobial susceptibility patterns of preformed C. albicans/S. gordonii mixed-species biofilms formed by the different parental and deletion mutant strains in BMM synthetic saliva (using monotherapy with either amphotericin B or clindamycin. Drugs were added at the following concentrations: clindamycin at 100, 10, 1, 0.1, and 0.01 μM, and amphotericin B at 16, 4, 1, 0.25, and 0.0625 μg/ml. Error bars represent standard errors of the means. Download FIG S4, PDF file, 0.2 MB. Copyright © 2019 Montelongo-Jauregui et al. 2019 Montelongo-Jauregui et al. This content is distributed under the terms of the Creative Commons Attribution 4.0 International license.
