# Supplementary figures and images for: Contributions of Candida albicans Dimorphism, Adhesive Interactions, and Extracellular Matrix to the Formation of Dual-Species Biofilms with Streptococcus gordonii
Source: mBio. 2019 Jun 18;10(3):e01179-19. doi: 10.1128/mBio.01179-19 (PMC6581863; doi:10.1128/mBio.01179-19)

**Figure S1.**

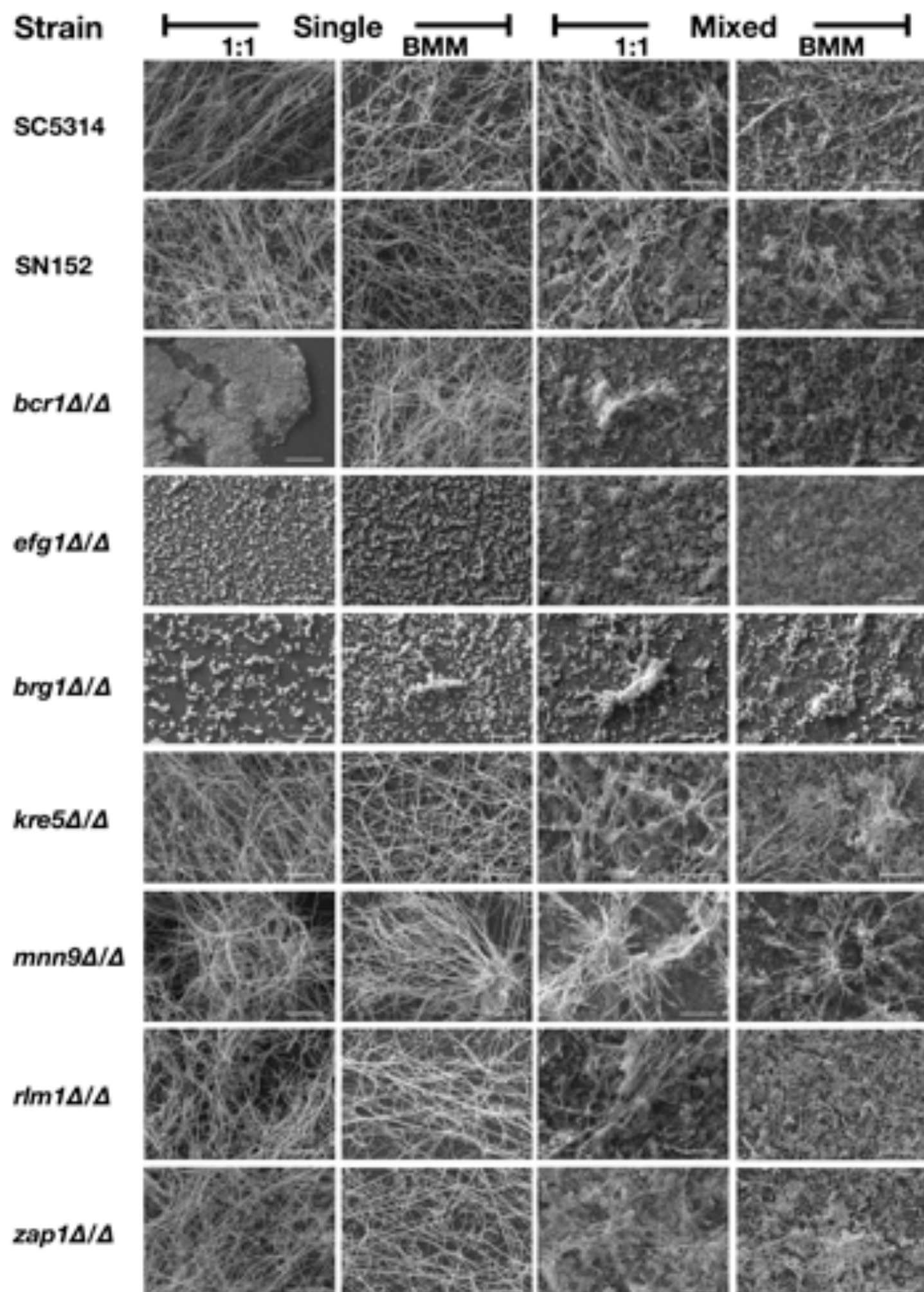

Supplement: FIG S1 [file mBio.01179-19-sf001.pdf]

Figure S2.

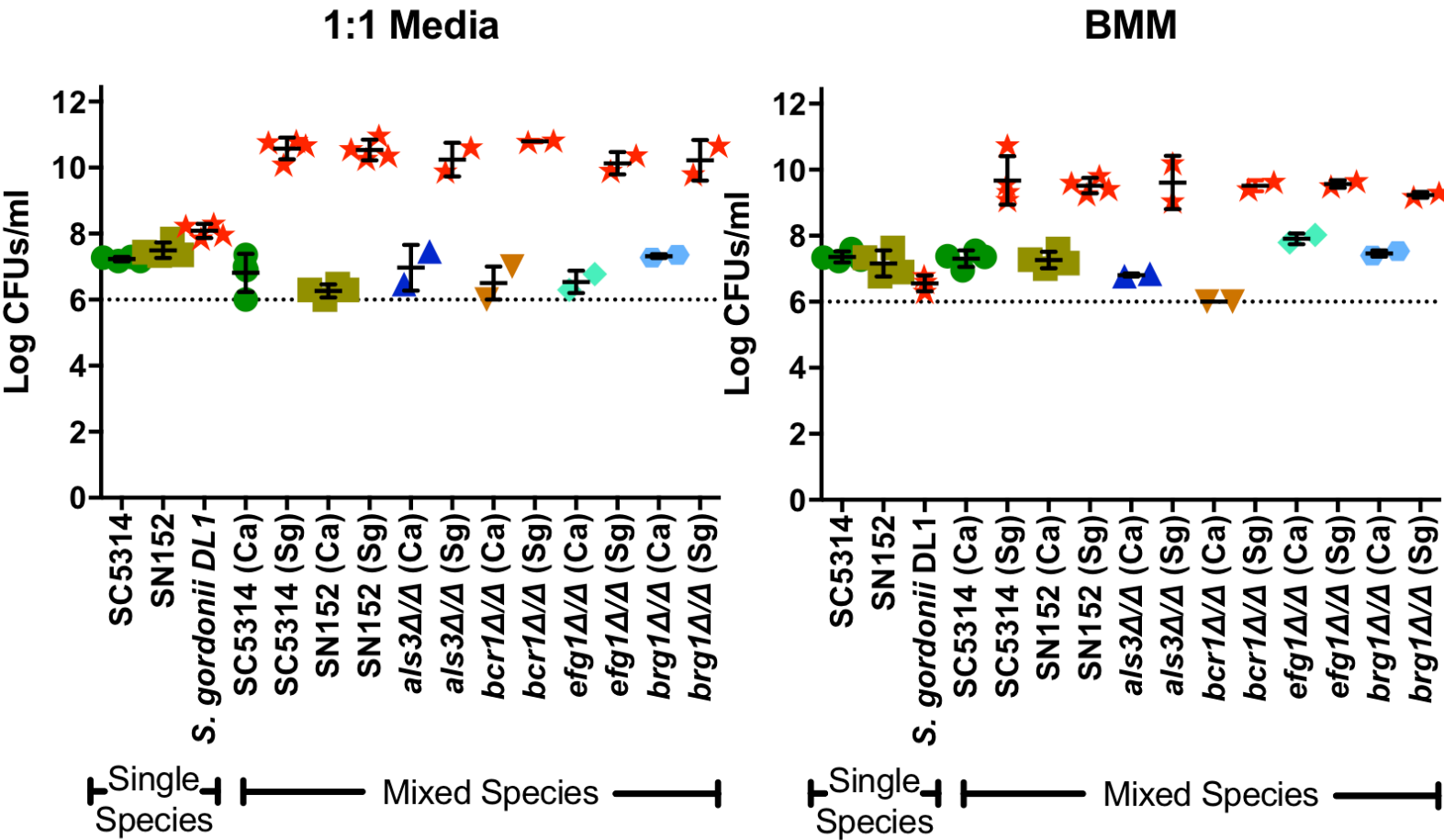

Supplement: FIG S2 [file mBio.01179-19-sf002.pdf]

Figure S3.

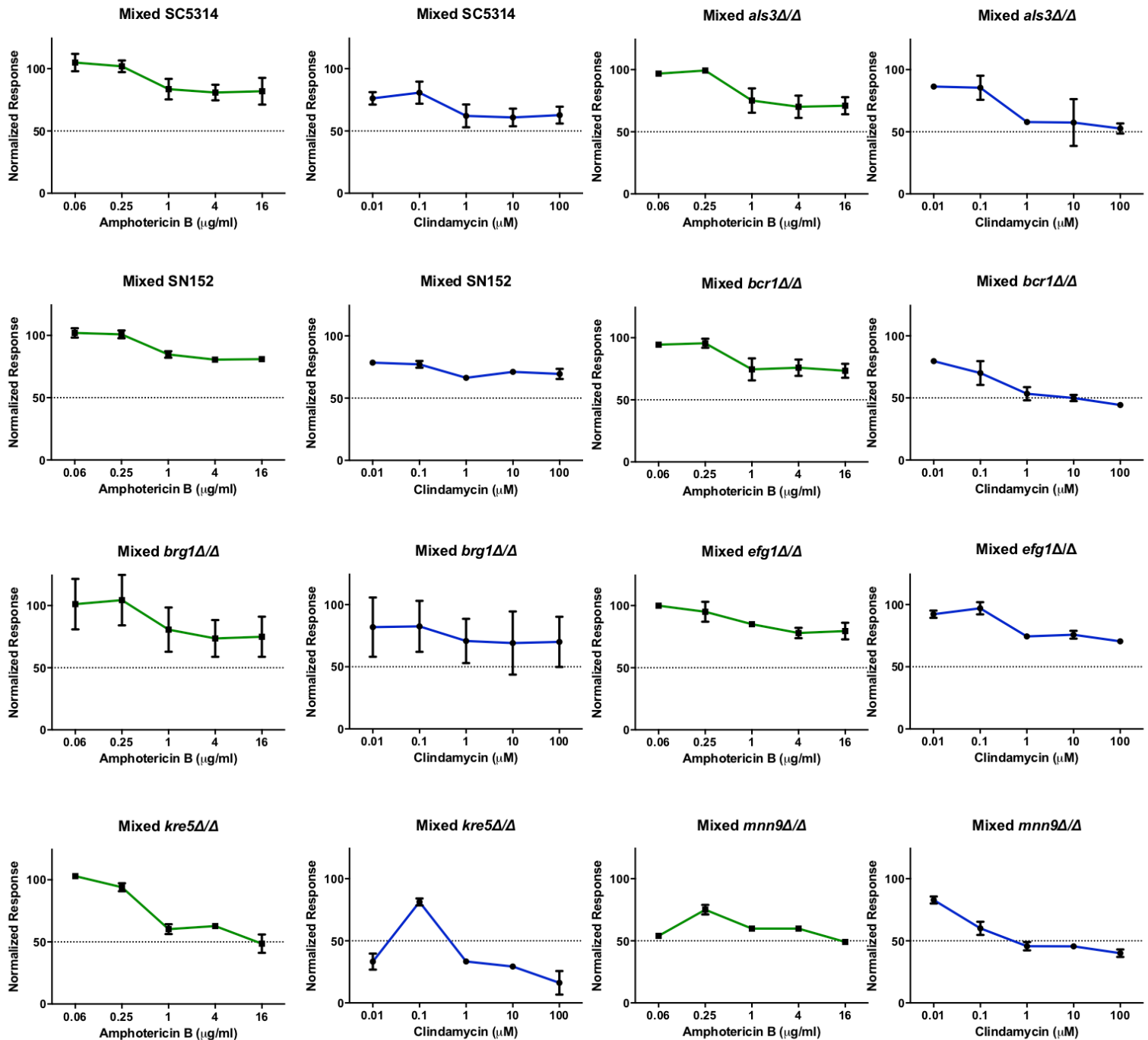

Supplement: FIG S3 [file mBio.01179-19-sf003.pdf]

Figure S4.

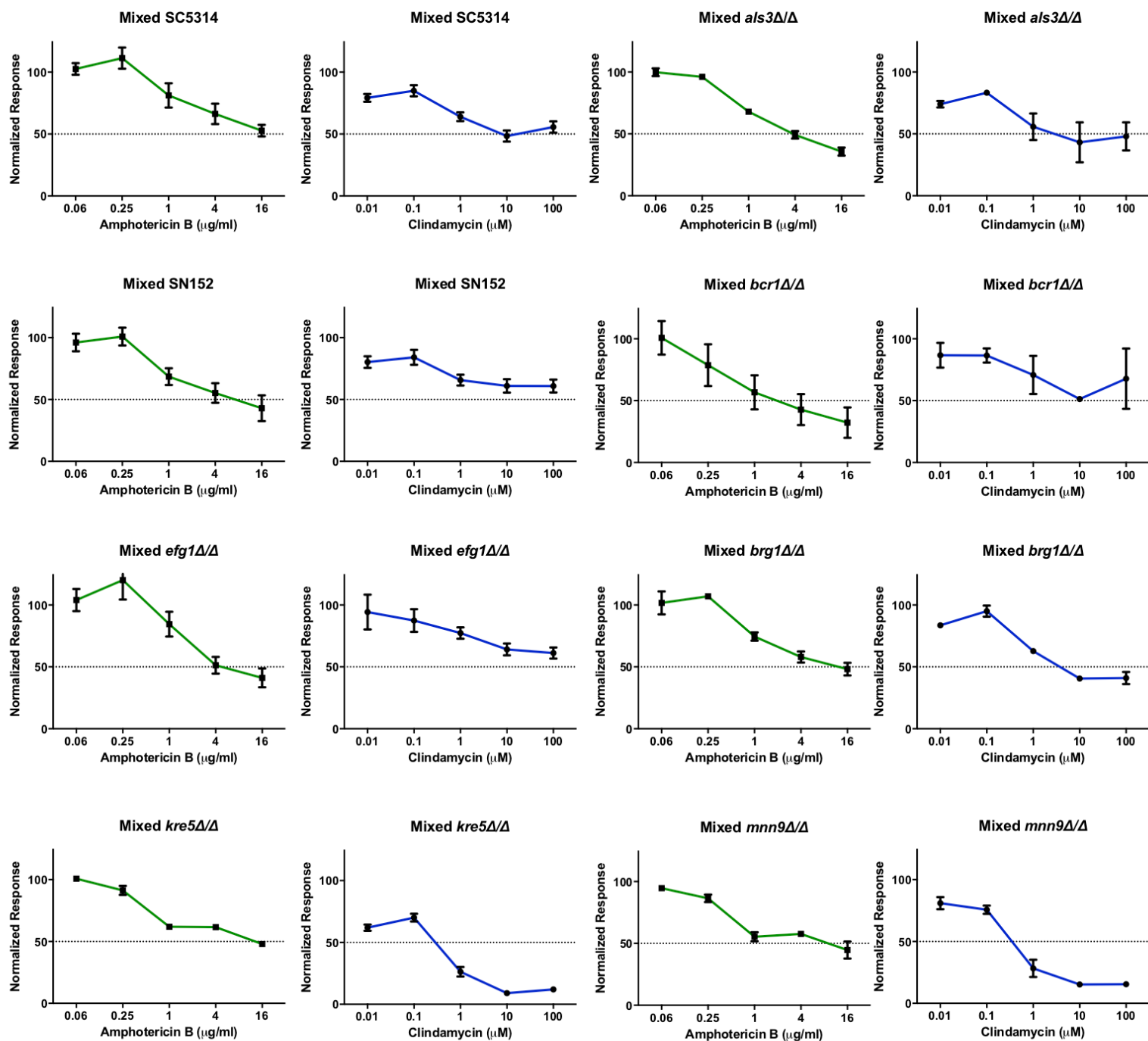

Supplement: FIG S4 [file mBio.01179-19-sf004.pdf]
